# The magnitude of anemia and associated factors among pregnant women attending public institutions of Shire Town, Shire, Tigray, Northern Ethiopia, 2018

**DOI:** 10.1186/s13104-018-3706-x

**Published:** 2018-08-17

**Authors:** Awoke Kebede, Hadgu Gerensea, Freweyni Amare, Yared Tesfay, Girmay Teklay

**Affiliations:** 1 School of Nursing, College of Health Sciences and Comprehensive Specialized Hospital, Aksum University, Aksum, Ethiopia; 2 Students Clinic, Aksum University, Shire Campus, Shire, Ethiopia

**Keywords:** Pregnant women, Anemia, Micronutrient deficiency

## Abstract

**Objective:**

Anemia is a widespread health problem among pregnant women causing maternal/infant morbidity and mortality mainly in low-income countries. Understanding of the magnitude of anemia and related socio-demographic variables in a specific setting would help scale-up preventive and therapeutic measures in a locality. So that this study focuses on the magnitude of anemia and its associated factor among pregnant women attending antenatal care in public hospitals of shire town and using institution based cross-sectional study design on 480 randomly selected study subjects.

**Result:**

The overall prevalence of anemia was 16.3%. Majority of the participants (52%) have mild anemia (10–10.9 gm/dl). Pregnant mothers with human immunodeficiency virus, intestinal parasitic infection and having lower inter-pregnancy gap were significant predictors of anemia. Preventing infection of the mother during pregnancy and making the gap between pregnancies are necessary.

## Introduction

Anemia is a condition in which the number or size of red blood cells and thus hemoglobin (Hgb) concentration fall below the required level resulting in the impaired capacity to transport oxygen [1]. Anemia is a major global public health concern. The highest proportions of peoples affected are from sub-Saharan Africa and Southeast Asia. It affects all ages- and sex-groups with the highest prevalence, 43%, 38%, occurring among less than 5 years children and pregnant women respectively [1].

Anemia in pregnancy is defined based on the level of pregnancy. For first and third trimesters Hgb levels < 11 g/dl and for second trimester < 10.5 g/dl are considered anemic [2]. It was estimated that anemia may be responsible for as much as 20% of all maternal deaths in sub-Saharan Africa through three main mechanisms [3]. And Ethiopian Demographic Health Survey (EDHS) report, 17% of reproductive age women were estimated to be anemic and 22% of the pregnant women are anemic [4].

Therefore the aim of this study was to determine the magnitude of anemia and associated factor among pregnant women attending the antenatal clinic in public health in shire town.

## Main text

### Study area

The study was done in Shire town northwestern zone which was located in north part of Ethiopia 1087 km from Addis Ababa, and 300 km from Mekelle city, the Capital of Tigray with a total population of 47,284 (21,867 male and 25,417 female). Based on the 2007 national census conducted by the central statistical agency, this town has total population of 47,284, of whom 21,867 are men and 25,417 women. Majority of the inhabitants (85.11%) follow Ethiopian Orthodox Christianity but 14.67% are Muslims. The Suhul hospital found in shire town is estimated to serve more than one million people residing in the town and neighboring areas [5–12]. The study was conducted from April 1–30, 2018.

### Study design

The institutional based cross-sectional study design was conducted.

### Sample size

A total of 313 samples were calculated using a single population proportion formula by assuming 5% marginal error and 95% confidence interval (σ = 0.05) and prevalence of anemia 27.4% [13] and by adding 10% of non-response rate.

### Sampling technique

From all health facilities in the study area, one hospital and two health centers were selected. Random sampling method was used to select participants from women attending antenatal care follow-up.

### Data collection tool

A structured questionnaire was adopted and adapted from review of relevant literature and arranged according to particular objective it can address. Before actual data collection, the tool was pre-tested in St. Mary Hospital. Three days of training was given to all data collectors and supervisors prior to pretesting. Eight data collectors who had completed a degree in midwives were recruited. The data was collected through self-administer questionnaire.

#### Variables

Dependent variable: Magnitude of anemia

Independent variables: Age, education, gravida/parity, gestational age, medical/surgical problems (intestinal parasite, HIV, malaria, malnutrition)

### Data processing and analysis

Data were entered and analyzed using SPSS version 22.0. Descriptive statistics were employed to calculate frequencies and display findings. Association was measured using binary logistic regression. Based on Bivariate analysis variables that showed significant association at (p < 0.25) were entered into the multivariable analysis to select Predictor variables of factors affecting anemia in pregnant women. The final model were then tested for its goodness of fit by Hosmer and Lemeshow p-value and p-value > 0.05 was the best fitted. Finally, variables that showed significant associations at (p < 0.05) were identified as independent predictors of affecting anemia in pregnant women.

### Ethical considerations

The Ethical approval was approved by the Institutional Review Board (IRB) of the College of Health Sciences, Aksum University. Communications with the health center administrations were made through a formal letter obtained from Aksum University, College of Health Science and TRHB. The objective and importance of the study were explained to the study participants. Data was collected after full informed written consent was obtained from participants aged 18 years and more, but age less than 18 years from the guardian. Confidentiality of the information was maintained throughout by excluding names as identification in the questionnaire and keeping their privacy during the interview by interviewing them alone.

## Results

### Socio-demographic data

A total of 480 pregnant women aged 18–38 years (mean age 28.2) were examined during the study period. The majority (73.3%) of the women were within the age group 25–31. The majority (89.0%) of the participants were married. More than half of the participants (52.2%) were unemployed. The medical history of the participants showed that 35 (7.3%) had clinical malaria within past 1 year, 35 (7.3%) intestinal parasitic infections (IPIs) and 31 (6.5%) reactive to HIV antibody test. Although the majority of the participants 343 (71.5%) had a normal body mass index, there were 71 (14.8%) underweight and 66 (13.8%) overweight individuals. While 25 (5.2%) of the participants were self-reported alcohol consumers, slightly more 31 (6.5%) were smokers. All the women answered that they had no history of recent bloody diarrhea or bleeding, chronic kidney or other infectious or non-infectious diseases (see Table 1).

**Table 1:** Socio-demographic characteristic and behaviors of pregnant mothers attending ANC in public institutions of Shire Town, Tigray, Northern Ethiopia, 20,168 (N = 480)

Variable	Anemia status		Total no. (%)
	Anemic, no. (%)	Non-anemic, no. (%)	
Age in year			
18–24	6 (22.2)	21 (77.8)	90 (5.6)
25–32	57 (16.1)	295 (83.9)	352 (73.3)
32–38	15 (14.9)	86 (85.1)	101 (21.0)
Marital status			
Single	6 (11.3)	47 (88.7)	53 (11.0)
Married	72 (16.9)	355 (85.1)	427 (89.0)
Employment			
Employed	30 (13.2)	198 (86.8)	228 (47.5)
Unemployed	48 (19.1)	204 (80.9)	521 (52.5)
Malaria during pregnancy			
Yes	11 (31.4)	24 (68.6)	35 (7.3)
No	67 (15.1)	378 (84.9)	445 (92.7)
Intestinal parasite			
Yes	13 (37.1)	22 (62.9)	35 (7.3)
No	65 (14.6)	380 (85.4)	445 (92.7)
HIV status			
Positive	12 (38.7)	19 (61.3)	31 (6.5)
Negative	66 (14.7)	383 (85.3)	449 (93.5)
Smoking			
Yes	9 (29.0)	22 (71.0)	31 (6.5)
No	69 (15.4)	380 (84.6)	449 (93.5)
Alcohol consumption			
Yes	10 (40.0)	15 (60.0)	25 (5.5)
No	68 (14.9)	387 (86.1)	455 (94.5)

Regarding trimester, most participants (41.3%) the current pregnancy was their first (primigravida) and for 155 (32.3%) it was their fourth or above (multi-gravida), 127 (26.5%) were experiencing their second or third pregnancy. Most of the participants 189 (39.9%) were in their first trimester followed by those in their third 167 (34.8%) and in their second 124 (25.8%). Regarding the inter-pregnancy gap, for those who have more than one pregnancy, 32 (12.1%) had below 2, 143 (50.7%) 2–3 and 105 (37.2%) had more than 3 years of gaps between their successive pregnancies.

The overall prevalence of anemia (Hgb < 11.0 g/dl) among the pregnant women was 16.3% with 48% moderate and 52% mild cases none being severe. The mean Hgb was 12 g/dl with the lowest and highest values 7.8 and 13.8 g/dl respectively. The mean hematocrit was 33.7%. The prevalence of anemia was high among pregnant women who were IPIs-positive (37.1%), HIV-seropositive (38.7%) and mothers with more than three pregnancies (32.3%) and prevalence of anemia increased as the number of pregnancy increased (see Table 2).

**Table 2:** Pregnancy-related conditions of anemia in pregnant mothers attending ANC in public institutions of Shire Town, Tigray, Northern Ethiopia, 20,168 (N = 480)

Variable	Anemia status		Total no. (%)
	Anemic, no. (%)	Non-anemic, no. (%)	
Trimester			
First	30 (15.9)	159 (84.1)	189 (39.4)
Second	22 (17.7)	102 (82.3)	124 (25.8)
Third	26 (15.6)	141 (84.4)	167 (34.8)
No. of pregnancy			
1 (current)	24 (12.1)	174 (87.9)	198 (41.3)
2–3	18 (14.2)	109 (85.8)	127 (26.5)
≥ 4	36 (23.2)	119 (76.8)	155 (32.3)
Inter-pregnancy gap (years)			
**< **2	6 (14.7)	29 (85.3)	34 (12.1)
2–3	22 (15.4)	121 (84.6)	143 (50.7)
≥ 4	14 (13.3)	91 (86.8)	105 (37.2)
Body mass index			
Under weight	19 (19.7)	57 (80.3)	71 (14.8)
Normal	50 (14.6)	293 (85.4)	343 (71.5)
Overweight	14 (21.2)	52 (78.8)	66 (13.8)

### Factors associated with anemia

In multivariate analysis, participants who are positive for intestinal parasite were 3.46 times more likely to have anemia than those who were not (OR 3.46, 95% CI 1.67–7.20, p = 0.001). Similarly, HIV-positivity was a statistically significant predictor of anemia (OR 3.67, 95% CI 1.70–7.90, p = 0.001). Individuals who had less than 2 years inter pregnancy gap were also at significantly higher risk of having anemia than those who had 2–3 years gaps (OR 7.312, 95% CI 3.041–17.587, p = 0.001). Finally, all the above three variables positive for an intestinal parasite, HIV positive and narrow inter pregnancy gap were found to be independently associated with the occurrence of anemia in the multivariate model (see Table 3).

**Table 3:** Factors associated with anemia in pregnant mothers attending ANC in public institutions of Shire Town, Tigray, Northern Ethiopia, 20,168 (N = 480)

Variable	Anemia status		COR (95% CI)	p-value	AOR (95% CI)	p-value
	Anemic, no. (%)	Non-anemic, no. (%)				
Age in year					–	
18–24	6 (22.2)	21 (77.8)	3.24 (1.470–7.133)	0.04		
25–32	57 (16.1)	295 (83.9)	2.27 (0.931–5.536)	0.72		
32–38	15 (14.9)	86 (85.1)	1.00			
Malaria during pregnancy					–	
Yes	11 (31.4)	24 (68.6)	2.58 (1.210–5.526)	0.14		
No	67 (15.1)	378 (84.9)	1.00			
HIV status						
Positive	12 (38.7)	19 (61.3)	3.67 (1.700–7.904)	0.001	2.802 (1.174–6.635)	0.02
Negative	66 (14.7)	383 (85.3)	1.00			
Intestinal parasite						
Yes	13 (37.1)	22 (62.9)	3.46 (1.670–7.200)	0.001	3.340 (1.49–7.48)	0.003
No	65 (14.6)	380 (85.4)	1.00			
Smoking					–	
Yes	9 (29.0)	22 (71.0)	2.25 (1.995–5.090)	0.05		
No	69 (15.4)	380 (84.6)	1.00			
Alcohol consumption					–	
Yes	10 (40.0)	15 (60.0)	2.79 (1.637–6.794)	0.06		
No	68 (14.9)	387 (86.1)	1.00			
No. of pregnancy					–	
1 (current)	24 (12.1)	174 (87.9)	0.83 (0.433–0.961)	0.59		
2–3	18 (14.2)	109 (85.8)	0.613 (0.301–0.804)	0.07		
≥ 4	36 (23.2)	119 (76.8)	1.00			
Inter-pregnancy gap						
**< **2 years	6 (14.7)	29 (85.3)	7.31 (3.041–17.587)	0.001	6.665 (2.69–16.483)	0.001
2–3 years	22 (15.4)	121 (84.6)	1.182 (0.573–2.436)	0.65		
≥ 4 years	14 (13.3)	91 (86.8)	1.00			

*AOR* adjusted odds ratio, *CI* confidence interval, *COR* crude odds ratio, *HIV* human immunodeficiency virus

## Discussion

The overall anemia prevalence among the study participants was 16.3%. This result is comparable with the results from Mekelle (16.6%) and Gonder (19.3%) respectively [14, 15]. But this finding is lower than findings of Gilgel Gibe (53.9%) and Gode South Ethiopia (565) [16, 17] and higher than in Iran (4.7%) [18]. The variations may be explained by factors such as malaria endemicity particularly in Ghibe and Gode, study design and sample size, socio-economic and other baseline characteristics of the study populations as well as altitudinal differences. Further, early antenatal care follow-up and better healthcare awareness among the participants might have contributed towards the observed differences in anemia prevalence. Also, in countries like Iran iron and folate supplements are routinely prescribed for pregnant women and that might be among the reasons for the apparent very low prevalence rate in the country.

Positivity for intestinal parasite was significantly associated with anemia. The result of the study was consistent with the literature on possible factors of anemia in pregnancy in Shalla Woreda of Oromia region In Ethiopia [19]. This is expected as intestinal parasites, apart from their competition for nutrients, are known to cause blood loss, loss of appetite reduced motility of food through the intestine and damage to the wall of the intestine leading to mal-absorption of nutrients.

The study also showed a significant association between anemia and HIV. Pregnant women with HIV was more likely to have anemia than those who without HIV in agreement with previous reports [20]. The high prevalence of anemia in HIV-positive pregnant women might be due to the characteristics of the virus which results in increased metabolic and nutritional needs; poor intake of iron and other nutrients due to reduced appetite, mal-absorption of nutrients; and direct suppression of red blood cell production in the body [21].

The study result showed the prevalence of anemia increased as the number of pregnancy increased. This is in similar with the finding of Wolita Sodo, South Ethiopia [22] in which number of pregnancy had a statistically significant association with anemia. This could be explained the body will have enough time to recover from a nutrient loss that resulted from the previous pregnancy.

## Conclusion

The prevalence of anemia in the study area was low and HIV status, intestinal parasite, and inter-pregnancy gap were variables significantly associated.

### Recommendation

Although the condition appears mild as per the WHO criterion awareness creation on prevention of HIV, personal hygiene and environmental sanitation to control intestinal parasite infection, and use of contraceptives to widen the inter-pregnancy gap are important to further prevent pregnancy-related anemia. Since the present investigation was conducted on a relatively smaller small sample size, studies using a larger sample size are needed to better understand the scale of anemia among pregnant women in the study area.

## Limitation

Smaller sample size and the nature of cross-sectional study which does not show cause and effect relationship.

## Abbreviations

IPIs: intestinal parasitic infections
WHO: World Health Organization
Hgb: hemoglobin
EDHS: Ethiopian Demographic Health Survey
HIV: human immunodeficiency virus
